# Intracerebral Electrical Stimulation of the Left Occipito-Temporal Cortex Induces Pure Alexia

**DOI:** 10.1162/NOL.a.205

**Published:** 2025-12-18

**Authors:** Marion Marchive, Luna Angelini, Aliette Lochy, Louis Maillard, Sophie Colnat-Coulbois, Bruno Rossion, Jacques Jonas

**Affiliations:** Université de Lorraine, CNRS, IMoPA, Nancy, France; Institute of Cognitive Science and Assessment, Université du Luxembourg, Esch-sur-Alzette, Luxembourg; Bioserenity, Paris, France; Psychological Sciences Research Institute, University of Louvain, Louvain-La-Neuve, Belgium; CHRU-Nancy, Université de Lorraine, Service de Neurologie, Nancy, France; CHRU-Nancy, Université de Lorraine, Service de Neurochirurgie, Nancy, France

**Keywords:** alexia, intracerebral electrical stimulation, reading, stereotactic electroencephalography (SEEG), visual word form area (VWFA)

## Abstract

The ability to read relies on the rapid mapping of perceived visual letters and their combinations (i.e., visual word forms) to phonology and meaning. The central role of the left ventral occipito-temporal cortex (VOTC) in processing letter strings, initially suggested by lesion studies, is now widely accepted. Although this brain region has been extensively studied with functional magnetic resonance imaging (fMRI), its causal role as a critical node of a cortical network for reading remains unclear. Here we report a comprehensive case of pure alexia during direct electrical stimulation (DES) of the left VOTC (patient SV, female, 38 yr old, implanted with intracerebral electrodes for refractory epilepsy). During DES of the left posterior occipito-temporal sulcus, but not of neighboring and remote cortical sites, SV was transiently impaired at reading single words while being able to slowly read letter-by-letter. However, SV was impaired when presenting a single letter in a rapid serial visual presentation, which showed that their letter reading is not entirely preserved. In contrast, DES to the same critical sites left performance for oral naming, auditory naming, reading numbers, writing, auditory lexical decision, and semantic matching of pictures unaffected. Intracerebral electrophysiological frequency-tagging investigations showed highly word-selective neural responses at the critical sites. These functional responses were abolished by concurrent DES, which also affected remote word-selective neural activity in the left VOTC. Altogether, these observations provide original evidence for word-selective representations of the left VOTC as a critical node of the cortical reading network.

## INTRODUCTION

Reading is a real challenge for the human brain, requiring association of complex variable visual patterns to sounds and meanings within a few hundred milliseconds. The question of specific brain regions for processing visual letter strings has long been raised (Dejerine, 1891). Brain lesions associated with pure alexia (i.e., reading impairment with preserved writing, naming, oral repetition, or auditory comprehension) revealed the critical role of the left ventral occipito-temporal cortex (VOTC) in reading (e.g., Cohen et al., 2003; Damasio & Damasio, 1983; Gaillard et al., 2006; Robotham et al., 2023; Starrfelt & Shallice, 2014; Starrfelt & Woodhead, 2021) with neuroimaging studies specifically defining a visual word form area (VWFA) in the posterior fusiform gyrus (e.g., Cohen et al., 2000, 2002; Gaillard et al., 2006; see also Petersen et al., 1990). Intense debates regarding the degree of specificity and function of this region have been raised (e.g., Caffarra et al., 2021; Price & Devlin, 2003; Yeatman & White, 2021), first and foremost about whether it processes only prelexical (Binder et al., 2006; Dehaene et al., 2002) or also lexical representations (Glezer et al., 2009, 2015). Moreover, the VWFA is described either as a single region including a hierarchical gradient from letters to words (e.g., Dehaene et al., 2005; Vinckier et al., 2007; see Caffarra et al., 2021, for review) or as decomposed into multiple discrete VOTC regions with their precise functional organization remaining elusive (e.g., White et al., 2019; Yablonski et al., 2024; see Caffarra et al., 2021, for review).

Neural investigations of reading have largely relied on functional magnetic resonance imaging (fMRI), which suffers from magnetic susceptibility artifacts, partially masking signals from the anterior VWFA portion (Wandell, 2011). In contrast, recordings in awake patients implanted with intracranial electrodes (i.e., intracranial EEG [iEEG]) provide direct measures of neural activity during reading without such artifacts, with high spatial and temporal resolution. iEEG studies generally support a hierarchical view of reading, with increasingly complex representations from letters to words along the VOTC/VWFA (Hirshorn et al., 2016; Lochy et al., 2018; Nobre et al., 1994; Thesen et al., 2012).

Importantly, iEEG also allows for direct electrical stimulation (DES), a powerful method for assessing the critical function of a targeted region (Borchers et al., 2012; Desmurget et al., 2013; Jonas & Rossion, 2023; Ojemann et al., 1989; Penfield, 1958). Cases of transient reading arrest during DES of the left VOTC have provided evidence that this region is critical for reading (Cloppenborg et al., 2021; Hirshorn et al., 2016; Mani et al., 2008; Sabsevitz et al., 2020; Woolnough et al., 2021, 2022; Woolnough & Tandon, 2024). However, reports have rather been anecdotal, that is, without quantitative analyses of behavioral effects and independent functional mapping (e.g., Mani et al., 2008; Sabsevitz et al., 2020), performed with only a few different reading tasks (e.g., Sabsevitz et al., 2020; Woolnough et al., 2022; Woolnough & Tandon, 2024), or only briefly described among extensive iEEG recording investigations (e.g., Hirshorn et al., 2016; Woolnough et al., 2021). Most importantly, in-depth evaluation of other language functions is lacking, so that clear cases of pure alexia following DES to the VWFA in the left VOTC remain elusive.

Here we report a comprehensive investigation of a rare case of pure alexia during DES of the VWFA. To clarify the critical role of the stimulated region, reading was tested with various tasks (i.e., nine reading tasks including reading words, letters, pseudowords presented at different speeds) along with nonreading tasks (i.e., writing, visual object naming, auditory object naming, semantic matching, auditory lexical task). Task performance was assessed quantitatively with accuracy rates and response times (RTs) before, during, and after stimulation. Functional iEEG mapping performed with a frequency-tagging approach provided objective quantification of word-selective neural activity (Lochy et al., 2015, 2018), overall demonstrating a tight relationship between behavior and independently measured category-selective neural processes in this key cortical region for reading.

## MATERIALS AND METHODS

### Case Description

The subject is a right-handed 38-year-old woman (SV) with refractory focal epilepsy who underwent stereotactic electroencephalography (SEEG) in January 2022 as part of the clinical investigation for her epilepsy. The SEEG showed a left anterior temporal epilepsy. Patient SV gave written consent for the experimental procedures that were administered during her SEEG exploration and were part of the clinical investigation. The study protocol was approved by a national ethics committee certified by the French Ministry of Health (Institutional Review Board: IORG0009855). She also gave written consent for the fMRI experiment (CARTA, N° IDRCB: 2021-A02968-33) and the use of video material.

### Neuropsychological Assessment

#### General assessment

SV had an average general intellectual efficiency (total IQ: 90; WAIS-IV [Wechsler, 2008]). Her neuropsychological assessment showed normal performance in verbal short-term memory (subtest Digit Span from WAIS-IV) but difficulties in the retrieval of long-term nonverbal and verbal information and memory for face identities (French adaptation from the Buschke Selective Reminder Test [Buschke, 1973; Rectem et al., 2004]; Brief Visuospatial Memory Test—Revised [Benedict, 1997]; Faces subtest of the MEM-III [Wechsler, 2001]). Executive and attentional functions were preserved although with difficulties in mobilizing attention (subtest Alertness, Sustained Attention, Divided Attention TAP [Zimmermann & Fimm, 2002]; subtest Stroop and Similarities from WAIS-IV). SV performance was below the expected range in the oral naming test (DO-80 naming test; Deloche & Hannequin, 1997) with a score of 64/80 (± −14.3 from the population norms mean: 79.02) and a total response time of 262 s (± −8.94 from the population norms mean: 100.92).

Her performance was below the normal range in verbal and nonverbal episodic memory and oral naming, consistent with an epileptic focus in the left anterior temporal lobe (Hamberger, 2015; Hermann et al., 1992; Hoppe et al., 2007; Mayeux et al., 1980).

#### Reading performance

Outside of the SEEG procedure, SV performed reading tests of a French battery for adults (Ecla 16+; Gola-Asmussen et al., 2010), which evaluated reading accuracy and speed (see Table S1 of the Supplementary Materials, available at https://doi.org/10.1162/NOL.a.205). Her performance was compared with the mean and standard deviation of a sample of 181 people for ECAL 16+ scores of the same socioeconomic level (Ecla 16+; Gola-Asmussen et al., 2010). For each task, a *Z* score was calculated by comparing SV’s accuracy to the mean accuracy of the population. SV was in the normal range for each task (performance less than 2 standard deviations of the mean population) except for reading letters (time: 22 s compared to 16.25 s ± 2.82 for the population). Additionally, she completed a reading-aloud isolated words test with varying lengths within a minute from the LMC-R battery (Khomsi, 1999) with 94 words correctly read out of 106.

### Stereotaxic Placement of Intracerebral Electrodes

**Figure 1.**
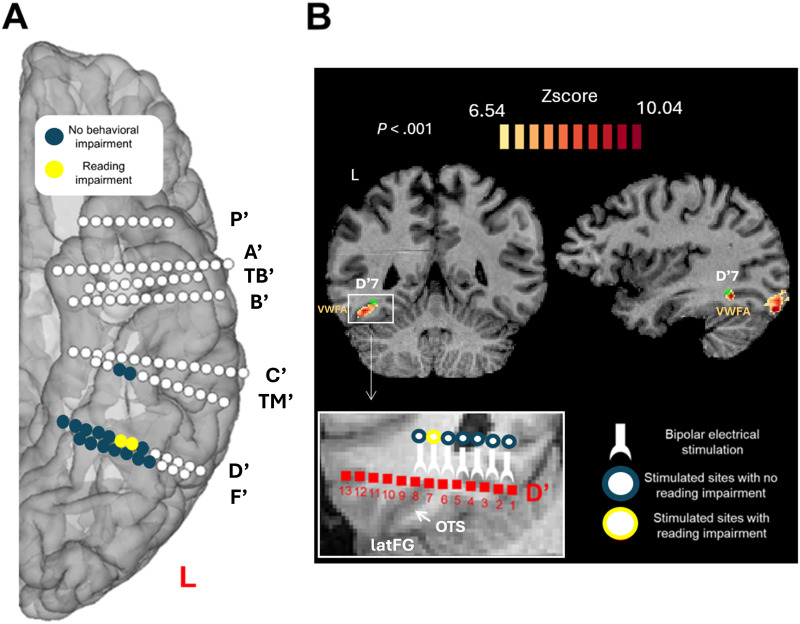
Anatomical location of the stimulation site inducing reading impairment. (A) Schematic localization of eight implanted electrodes, displayed using a reconstructed cortical surface of subject SV’s brain. Sites that were electrically stimulated with reading tasks are highlighted in color (yellow or blue). The critical site inducing transient pure alexia is displayed in clear colors (yellow) (D′6–D′7). (B) Top: the critical contact D′7, in green, is functionally located at the edge of the VWFA (activation for word − fixation cross, voxel *p* < 0.001 with Bonferroni correction). Bottom: critical contacts D′6–D′7 is anatomically located in the occipito-temporal sulcus (OTS), adjacent to the middle fusiform gyrus (lateral part of the fusiform gyrus; latFG).

Intracerebral electrodes (Dixi Medical, Besançon, France) were stereotaxically implanted into SV’s brain to delineate the seizure onset zone (Talairach & Bancaud, 1973). The sites of electrode implantation were determined based on noninvasive data collected during an earlier phase of the investigation. The implantation procedure is detailed in Salado et al. (2018). Fifteen electrodes were implanted in total, all targeting the left temporal lobe, with eight electrodes having contacts in the left VOTC (Figure 1). Electrode D′ (13 contacts) targeted the left lingual gyrus by crossing the occipito-temporal sulcus (OTS) and the left middle temporal gyrus (Figure 1).

The SEEG signal was recorded at a 512 Hz sampling rate on a 256-channel amplifier (Micromed). The reference electrode during data acquisition was a midline prefrontal scalp electrode (Fpz).

### Intracranial Electrical Stimulations

#### General procedure

DES was applied from 1 to 1.8 mA between two adjacent electrode contacts as biphasic square wave electrical pulses with 1,050 *μ*s width (alternating positive and negative 500 *μ*s phases, spaced from each other by 25 *μ*s) delivered at 55 Hz during 10 s (except for 10 out of 38 stimulations for D′6–D′7, and 9 out of 11 stimulations on other contacts performed during 5 s; see Table S2 in the Supplementary Materials). These stimulation parameters are typical in SEEG (Isnard et al., 2018).

During DES sessions, SV was asked to perform several types of tasks exploring different linguistic processes. Considering the limited amount of testing time afforded by the clinical context, we first identified the relevant electrode contacts for reading word impairment using one task (i.e., reading isolated words) and then further tested these contacts with complementary tasks.

The stimulation sites, the number of stimulation sessions performed at each stimulation site, and the type of task used for reading assessment are presented in Table S2 in the Supplementary Materials. The neurologist performed all electrical stimulations and set the stimulation site, the stimulation parameters, the task, and the onset of the stimulation. After identifying the most critical contacts for reading (D′6–D′7; see Results), the stimulation intensity was set at the beginning of each half-day of stimulation sessions at the minimal intensity leading to a reading impairment when stimulating contacts D′6–D′7 with the reading isolated task (half-day No 1: 1.2 mA; No 2: 1.6 mA; No 3: 1.2 mA; No 4: 1.8 mA). Once the stimulation intensity was set, all the stimulations of the corresponding half-day were performed at this intensity (in total four stimulation sessions were removed from analyses because they did not reach the threshold of the day). The precise onset was predetermined before the beginning of the stimulation sessions (SV was not aware of the stimulation onset). For each stimulation and task, we measured the accuracy and the RTs on correct trials (when possible), before, during and after stimulation, by retrospectively rewatching the video recording. For statistical analyses, we compared, as in Volfart et al. (2023), accuracy during the stimulation time with the accuracy outside stimulation (i.e., before and after) across stimulation sessions for each site and task using chi-square tests (*p* < 0.05). For RTs, we compared performance outside and during stimulation using the two-tailed permutation test (1,000 permutations) and estimated 95% confidence intervals for the mean differences by bootstrapping with replacement.

#### Reading and lexical decision tasks

##### Reading aloud isolated words, syllables, or pseudowords.

SV was presented with lists of isolated words, syllables, or pseudowords, and she had to read them aloud one by one. SV was asked to start reading once the neurologist pointed to an item. Words were composed of frequent irregular and regular French common nouns, with variable length between five or six letters. Syllables were 28 syllables, frequently encountered in French. Pseudowords were pronounceable letter strings of five or six letters.

##### Reading a text aloud.

SV was asked to read aloud a text, “Le pollueur,” from ECLA 16+ (see Table S1 in the Supplementary Materials).

##### Reading letters in a rapid serial visual presentation (RSVP) mode.

SV was asked to read aloud isolated single letters quickly presented one by one. Trials started with a fixation cross for 100 ms, followed by a randomly selected letter on the center of a screen for 150 ms, then a mask (i.e., ####) displayed for 300 ms (i.e., 1 letter every 550 ms, or 1818 Hz). The whole task took ∼32 s. For each stimulation session, SV was presented with all 26 letters repeated twice.

##### Reading words during fast periodic visual stimulation (FPVS).

**Figure 2.**
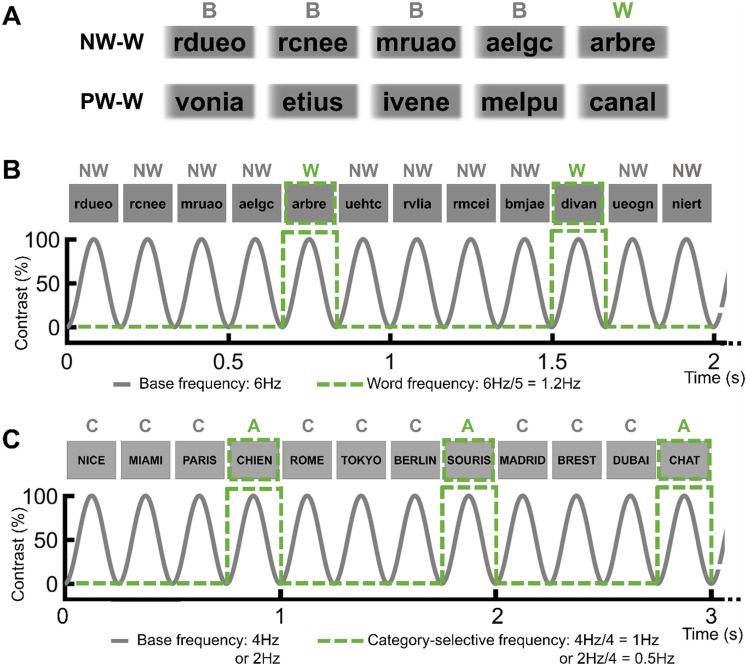
FPVS experiments. (A) Paradigm used to record word-selective neural activity (Lochy et al., 2015, 2016, 2018). Two types of sequences are used with base stimuli as nonwords (NW), pseudowords (PW), and oddball stimuli as words (W). Each sequence was presented randomly and repeated twice during one session. (B) Stimuli were presented by sinusoidal contrast modulation at 6 Hz. Words are inserted every five stimuli so that the frequency of the word-selective response is 1.2 Hz (i.e., 6 Hz/5). (C) Semantic Word paradigm used to measure semantic categorization (Volfart et al., 2021). Words are presented at 4 Hz, with an alternate category (i.e., name of animals) inserted every 4th stimulus (i.e., among city names), so that the frequency of the alternate category is 1 Hz (i.e., 4 Hz/4). A similar paradigm with a different presentation frequency (2 Hz) was also presented to the patient during the electrical stimulation of the critical site D′6–D′7 to study its effective connectivity.

SV was asked to read aloud isolated words presented quickly in an FPVS paradigm originally designed to record word-based semantic responses (SemWords; Volfart et al., 2021; Figure 2C), except that the frequency was reduced to 2 Hz (instead of 4 Hz), hence one word every 500 ms. Sequences of 64 s were composed of written words of city names presented on the screen at 2 Hz while inserting periodically (every 4th image, 0.5 Hz) an animal name. SV was asked to read aloud the animal names only. These stimulation sessions were also used to study the effective connectivity of critical contacts (see below).

##### Lexical decision tasks of written stimuli.

SV was presented with two versions of a task where she was asked to indicate if a letter string was a word without reading it aloud. Forty stimuli, five to six letter strings, half words, and half pseudowords, were used. This task was either computerized with stimuli presented one by one on a screen, or performed manually with stimuli presented on paper sheets. For the computerized task, each trial started with a fixation cross centered on the screen with a gray background, the stimulus then appeared, until SV answered by pressing buttons on a keyboard. Since she systematically replied outside stimulation that the words “*divan*,” “*hymen*,” and “*damné*” were nonwords, these words were considered as unknown and excluded from our analyses. For the noncomputerized task, two letter strings (one word, one pseudoword) were presented side by side on a paper sheet and SV was asked to answer which was a word by pointing to one of the two items.

##### Lexical decision tasks on oral stimuli.

The neurologist read aloud either a word or a pseudoword, and SV was asked to tell if the spoken item was a word or not by responding “yes” or “no.”

##### Reading numbers.

SV was asked to read aloud one by one numbers composed of three digits presented on a paper sheet.

#### Other tasks

##### Visual object naming.

SV was asked to orally name pictures of objects (living and nonliving) presented one by one.

##### Auditory object naming.

SV was asked to orally name an object (living or nonliving) from a verbal cue (short sentence) read aloud by the neurologist. For example, “Which animal meows?” (*the cat*).

##### Writing.

SV was asked to write isolated words dictated by the neurologist. One error was made during stimulation for the word “décor” (written “décore”), but this mistake was not considered significant as she did not detect this spelling as incorrect when presented with the incorrect item after stimulation.

##### Semantic matching with images.

SV was asked to match a target image (presented centered at the top) with one of two other images (at the bottom) displayed on a paper sheet (one semantically related image and one distractor), by pointing out the semantically related image at the bottom.

### Intracerebral Mapping of Word-Selective Responses

Well-validated FPVS, or “frequency-tagging” (Norcia et al., 2015; Rossion et al., 2020), paradigms were used to define intracerebral word-selective (Lochy et al., 2015, 2018) and semantic categorization neural activity (Volfart et al., 2021).

### Stimuli and Procedure

During these FPVS paradigms, subject SV was asked to fixate a small cross presented continuously at the center of the screen and to detect (by key press) brief nonperiodic color changes of this fixation cross.

#### FPVS Word paradigm

Word-selective responses were recorded using continuous sequences of visual stimuli, either nonwords (NW) or pseudowords (PW), presented at 6 Hz through sinusoidal contrast modulation with words inserted every fifth item, so that the word presentation frequency was 1.2 Hz, that is, 6 Hz/5 (Figure 2A and 2B). Each sequence lasted 70 s, with 2 s of fade-in and 2 s of fade-out. Subject SV was presented with six sequences in each condition. Stimuli and procedure are similar to those described in previous studies (e.g., Lochy et al., 2018), except for the presentation frequency, and will not be detailed here.

#### Semantic Words (SemWords4Hz) paradigm

Sequences consisted of presenting names of cities (e.g., Berlin, Paris, Tokyo) at 4 Hz (i.e., 4 images per second) for 74 s, with animal names (e.g., elephant) periodically embedded every 4th image. Hence, the semantic categorization response frequency was 1 Hz (Figure 2C). SV was presented with 12 stimulation sequences in total.

### Analysis of Intracerebral FPVS Responses

Analyses were performed using the free software Letswave5 (https://www.letswave.org/), following previously established procedures (e.g., Hagen et al., 2020; Jacques et al., 2020; Lochy et al., 2018; Volfart et al., 2022).

Segments of SEEG corresponding to stimulation sequences were extracted, starting 2 s after the onset of the sequence (i.e., after the fade-in period) until ∼68 s for word-selective response and ∼72 s for semantic categorization (before stimulus fade-out) to contain an integer number of 2 Hz cycles (respectively, ∼66 s and ∼70 s). These sequences, acquired with a scalp reference electrode (Fpz), were re-referenced to a bipolar montage (vs. the signal recorded at the adjacent contact located laterally along the electrode). Sequences were averaged in the time domain separately for each condition to increase the signal-to-noise ratio (SNR), and the amplitude spectrum was computed for each contact using Fast Fourier Transform (FFT).

Selective responses significantly above noise level at the word stimulation frequency (1.2 Hz), semantic categorization frequency (1 Hz), and their harmonics were determined in each condition as follows: (i) the FFT spectrum was cut into segments of 50 bins centered at the frequency of interest and the harmonics under the base rate, that is, 1.2, 2.4, 3.6, 4.8, 7.2, 8.4, 9.6, and 10.8 Hz for word-selective response and 1, 2, 3 Hz for the semantic response; (ii) the amplitude values of FFT segments relative to the first harmonics before the base response (i.e., up to 6 Hz for the Word paradigm and 4 Hz for the Semantic Words paradigm, therefore 4 harmonics for Words and 3 for Semantic Words); (iii) the summed FFT spectrum was transformed into a *Z* score. *Z* scores were computed as the difference between the amplitude at the word frequency bin and the mean amplitude of 48 surrounding bins (25 bins on each side, excluding the two bins directly adjacent to the bin of interest, i.e., 48 bins) divided by the *SD* of amplitudes in the corresponding 48 surrounding bins. A contact was considered as showing a significant response in a given condition if the *Z* score at the frequency bin of interest exceeded 3.1 (i.e., *p* < 0.001, one-tailed: signal > noise).

The significant responses for the two paradigms were then quantified for each contact as the sum of harmonics. The number of harmonics included in the analysis (Word paradigm: 1.2 Hz and 7 harmonics, up to 10.8 Hz excluding the 5th that coincided with the base frequency; SemWords4Hz: 1 Hz and 2 harmonics, up to 3 Hz) was based on previous intracerebral FPVS studies (e.g., Jonas et al., 2016; Lochy et al., 2018) and the highest number of consecutive harmonics that showed a significant response across contacts (*z* score > 3.1; i.e., *p* < 0.001). Baseline-corrected amplitudes were computed as the difference between the amplitude at each frequency bin and the average of 48 corresponding surrounding bins (25 bins on each side, i.e., 50 bins, but excluding the 2 bins directly adjacent to the bin of interest, i.e., 48 bins).

### Effective Connectivity: Electrical Stimulations During FPVS

Intracerebral electrical stimulations were applied to the critical contacts D′6–D′7 during sequences of the Semantic Words (SemWords2Hz) paradigm.

These sequences were run in addition to the sequences performed without the DES described above. During the DES sequences, patient SV was asked to read aloud only the names of animals that appeared on the screen. For this reason, the frequency rate of word presentation was reduced (words presented at 2 Hz instead of 4 Hz).

SV was sitting in her hospital bed facing the computer screen placed 70 cm away from her face. DES sessions were performed as follows. A SemWords2Hz continuous sequence was launched, running for 74 s (including 2 s of fade-in and 2 s of fade-out so that the full contrast sequence lasted 70 s). A bipolar DES was manually triggered approximately 22 s after the onset of the sequence (i.e., at 20 s of full contrast) and lasted for 10 s (1.2 mA, biphasic square wave electrical pulses with 1,050 *μ*s width at 55 Hz). Then, the sequence continued to run for approximately 40 s, without stimulation. With this recently validated procedure (Angelini, Jacques, et al., 2024; Angelini, Volfart, et al., 2024), we performed three DES sessions.

#### Analysis of intracerebral FPVS responses before, during and after stimulation

SEEG recordings obtained during the 70 s full contrast sequences were divided into periods of 10 s (i.e., 2 periods before DES, pre1 and pre2, 1 stimulation period, stim, and 3 periods after stimulation, post1, 2, 3). Given that the DES was triggered manually, its onset varied slightly across stimulation sessions; on average, the DES was administered after 20.81 s (20.81 ± 0.69) of full contrast visual presentation.

These sequences were acquired with a scalp electrode (Fpz) as a reference channel and were then re-referenced to a bipolar montage, as described earlier for the non-stimulated sequences. FFT was applied to the 10 s segments, which were then averaged across stimulation sessions separately for each stimulation site and period (e.g., averaging all the Pre1 segments relative to the stimulations on D′6–D′7). These averaged FFTs were then cropped into segments of 20 bins centered at the base frequencies (2 Hz) and harmonics (2 Hz and 6 harmonics, up to 14 Hz). The number of harmonics included in the analysis was based on the highest number of consecutive harmonics that showed a significant response across contacts (*z* score > 3.1; i.e., *p* < 0.001).

The amplitude values of these FFT segments were then summed, and baseline-corrected amplitudes were obtained as the difference between the amplitude at each frequency bin and the mean amplitude of 18 corresponding surrounding bins (10 bins on each side, i.e., 20 bins, but excluding the 2 bins directly adjacent to the bin of interest, i.e., 18 bins). As these analyses are conducted on shorter segments compared to the other intracerebral FPVS responses recorded in this patient (i.e., 10 s segments vs. the complete FPVS sequences of 70 s), they are characterized by lower frequency resolution and cannot be conducted with the same number of bins used for the complete FPVS sequences.

#### Statistical analyses

In order to observe a putative amplitude modulation of electrical stimulation on the frequency-tagged neural response throughout the brain, we focused on the comparison between the DES period and the average of the two segments obtained before DES (Pre1 and Pre2: PreGA).

The analysis of the FPVS sequences run during DES was limited to the contacts that showed a significant base response (*z* score > 3.1; i.e., *p* < 0.001) during the SemWords4Hz sequences recorded outside of stimulation.

To examine the amplitude modulation of the base response at a single contact/region level, we computed the amplitude decrease for each contact by subtracting the FFT spectra of the stimulation period from the average of Pre1 and Pre2 (PreGA minus Stim); we then transformed the result into a *Z* score (difference between the amplitude at each frequency bin and the mean amplitude of the corresponding 18 surrounding bins, divided by the standard deviation of amplitudes of these 18 bins). A contact was considered as showing a significant amplitude reduction during stimulation if the *Z* score exceeded 2.32 (*p* < 0.01; Angelini, Jacques, et al., 2024; Angelini, Volfart, et al., 2024).

### fMRI Word-Sensitivity

To localize the word-selective regions, SV performed an additional fMRI word localizer a few months after the SEEG procedure, with an adaptation of a block design word localizer paradigm (e.g., Dehaene et al., 2010), including two runs.

#### Word localizer

We adapted stimuli of previous word localizer studies (e.g., Amalric & Dehaene, 2016; Dehaene et al., 2010; Vinckier et al., 2007; Zhan et al., 2023). A standard block-design task was presented across two runs, each run consisting of seven blocks (18 s) repeated twice, one for each of seven different stimulus categories (i.e., words, faces, bodies, houses, tools, numbers, false fonts). All images within each category were randomly presented during 800 ms in black and white with a gray font and were centered on the screen with an interstimulus interval of 200 ms. A fixation cross was displayed between blocks for 9 s. Blocks were pseudorandomized within each run. Each run lasted 387 s, which corresponded to 258 dynamics. To ensure attention during the task, the subject had to press a button when an image was presented in red.

#### MRI acquisition

The data was acquired at the CIC-IT (University Hospital of Nancy). We acquired the MRI images using a 3T Siemens Magnetom Prisma system (Siemens Medical System, Erlangen, Germany) with a 64-channel head-neck coil. Anatomical images were collected using a high-resolution T1-weighted magnetization-prepared gradient-echo image (MPRAGE) sequence (192 sagittal slices, TR = 2,300 ms, TE = 2.6 ms; flip angle (FA) = 9°, field of view (FOV) = 256 × 256). Functional images were collected with a T2*-weighted simultaneous multislice echo planar imaging (SMS EPI) sequence (TR = 1,500 ms, TE = 30 ms, FA = 72°, FOV = 240 × 240 mm^2^, matrix size = 96 × 96, interleaved), which acquired 44 oblique-axial slices covering the entire temporal and occipital lobes. The total duration of each scan session was approximately 397 s, including 10 s of dummy scans. Images were back-projected onto a projection screen by an MRI-compatible LCD projector. SV observed the sequences through a mirror placed within the radio frequency head coil. The images subtended a viewing angle of 8° × 8° (33.4 cm × 33.4 cm) at a view distance of 240 cm. The experiment was conducted using the software E-prime 3.0 (Schneider et al., 2012).

#### Preprocessing analysis

Analysis and visualization were performed in BrainVoyager software (Goebel et al., 2006). A 3D motion correction (2 × 2 × 2 voxels) and a temporal high-pass filter were applied to functional runs and then co-registered to the anatomical scan, corrected with an intensity inhomogeneity correction. We then normalized anatomical and functional runs into the same anterior commissure–posterior commissure space. A general linear model contrast of words > fixation cross was applied to both runs to localize word-selective regions. The data were *z* normalized and reported with a Bonferroni correction at *p* < 0.001.

## RESULTS

**Figure 3.**
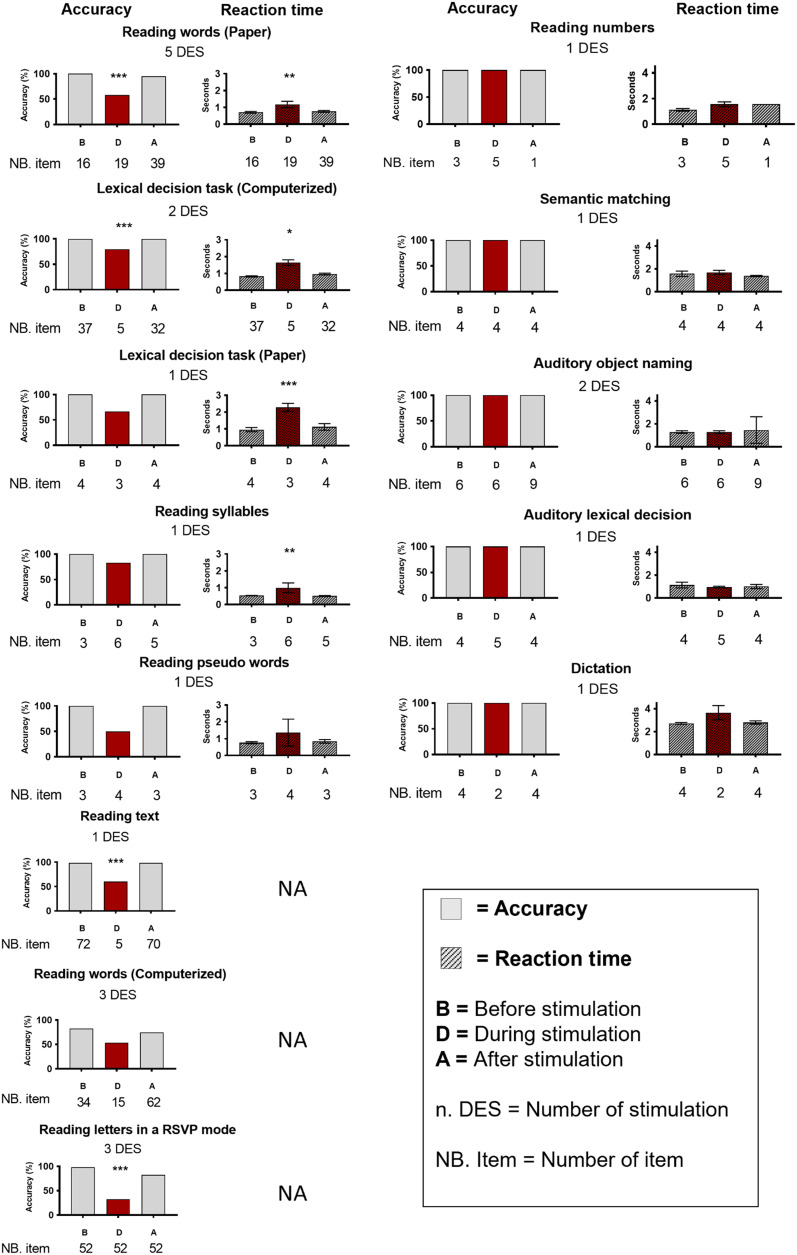
Accuracy and response times at all reading and language tasks while electrically stimulating word-selective contacts D′6–D′7. Impaired and nonimpaired tasks are shown on the left side and on the right side, respectively. For each task, the number of stimulation sessions and the number of trials across stimulation before (B), during (D) in red, and after (A) stimulation are indicated. Stars indicate a significant difference between during and outside (before and after) stimulation, ***p < 0.001, **p < 0.01, *p < 0.05. The total number of items presented is indicated under each task as “NB. item” before, during, and after the stimulation. The number of direct electrical stimulations is indicated under the name of each task as “DES.”

Accuracy and mean RTs are reported in Figure 3 for reading and nonreading tasks during stimulation sessions on D′6–D′7 contacts (see also Figure S7 in the Supplementary Materials). All statistical analyses of SV’s performance during DES on D′6–D′7 are indicated in Table S3 in the Supplementary Materials.

### Stimulating the Left Occipito-Temporal Sulcus Elicits Word Reading Impairment

Upon stimulation of a specific pair of contacts in the left OTS (bipolar stimulation of D′6–D′7; Talairach coordinates D′6: *x* = −29, *y* = −48, *z* = −14; D′7: *x* = −32, *y* = −50, *z* = −14; Figure 1), SV was transiently impaired at reading. When asked to read isolated words, her accuracy dropped suddenly (unread words) during stimulation and her RT increased (accuracy outside stimulation: 96.3%, during stimulation: 58%, (*χ*(1) = 17.881, *p* < 0.001; RTs: during = 1,174.54 ms > outside = 744.90 ms, *p* = 0.002, 95% CI [−825.93, −110.84], two-tailed permutation test; see Figure 3; see also Table S3 in the Supplementary Materials). Stimulating other contacts in the left VOTC (*N* = 11) did not impair word reading (see Figure 1; see also Table S2 in the Supplementary Materials). When reading a text, she read fluently, but as soon as the stimulation of D′6–D′7 was launched, she was unable to read some words and misread others (“Rome” instead of “Berlin”; accuracy outside stimulation: 98%, during stimulation: 60%, *χ*(1) = 21.175, *p* < 0.001; see Figure 3).

When asked to read words at a relatively fast rate, that is, at 0.5 Hz (animal names of the SemWords2Hz paradigm), her accuracy decreased during stimulation, with a trend toward significance (accuracy outside stimulation: 77%, during stimulation: 53%, *χ*(1) = 3.791, *p* = 0.052; see Figure 3). During this task, SV was also asked to raise her hand if she experienced a subjective difficulty in reading. SV raised her hand concomitantly to the stimulation for two out of three stimulation sessions (Supplementary Materials, Video S1). These stimulations were also used to study the effective connectivity of the D′6–D′7 critical site.

SV was also asked to read aloud isolated syllables and pseudowords. For both tasks, her performance decreased during stimulation (accuracy decrease and RTs increase) without reaching significance (except for RTs for syllables RTs: during = 992 ms > outside = 515 ms, *p* < 0.01, 95% CI [−1075.50, −88.50], two-tailed permutation test), probably because of a low number of trial sessions (only one stimulation session possible for each task).

For all the above tasks, SV was fully aware of her reading impairment during stimulation (“I had difficulty reading”). She never reported visual changes in the words or visual hallucinations. To assess whether her reading impairment concerns only full words or also isolated letters, SV was asked to read letter by letter whenever she was unable to read a word. She was able to correctly read letter by letter six out of seven unread words during stimulation (across 4 stimulation sessions). SV stated: “I can read the letters, but I can’t make out the word” (Supplementary Materials, Video S2) “I can’t link them [letters] together.”

Stimulating D′6–D′7 contacts during lexical decision tasks further showed that SV was impaired at distinguishing between pseudowords and meaningful words. SV was incorrect for two trials during stimulation, one during the two-alternative forced-choice paper sheet version (no response) and one during the computerized version (a word was classified as a pseudoword). Therefore, her accuracy at pointing to the real word decreased during stimulation (paper sheet: 66% during and 100% outside stimulation, *χ*(1) = 2.933, *p* = 0.087; computerized: 80% during and 100% outside stimulation, *χ*(1) = 13.989, *p* < 0.001). She also showed a significant increase in response time (paper sheet: RTs: during = 2,280 ms > outside = 1,040 ms, *p* < 0.001, 95% CI [−1612.50, −860], two-tailed permutation test; computerized: RTs: during = 1,638.75 ms > outside = 888.44 ms, *p* = 0.02, 95% CI [−1045.63, −460.36], two-tailed permutation test).

In contrast, SV was not impaired at lexical decisions when words and pseudowords were read aloud by the experimenter (see Figure 3; see also Table S3 in the Supplementary Materials), suggesting a selective deficit for lexical discrimination only when words were presented visually.

Stimulation of contacts D′6–D′7 did not affect reading numbers, semantic matching with images, visual and auditory naming, and writing under dictation (see Figure 3; see also Table S3 in the Supplementary Materials).

### Increasing the Presentation Speed Reveals a Single Letter Reading Impairment

Although SV was able to read letter by letter, she was usually slow and hesitant. Therefore, we designed a task in which she had to name letters at a fast rate in an RSVP mode (1.818 Hz; Supplementary Materials, Video S3). Across three stimulation sessions of D′6–D′7, SV performed well before stimulation (51/52 letters, 98%), but her performance dropped during stimulation (17/52, 32%). After stimulation, her performance increased without reaching the same level as before stimulation, especially because she was still impaired the following seconds after the end of stimulation (Figure 3). SV’s accuracy was significantly higher outside stimulation than during stimulation (*χ*(1) = 49.111, *p* < 0.001). SV did not report any perceived distortions of the letters.

### The Reading Critical Site Is Highly Word-Selective

**Figure 4.**
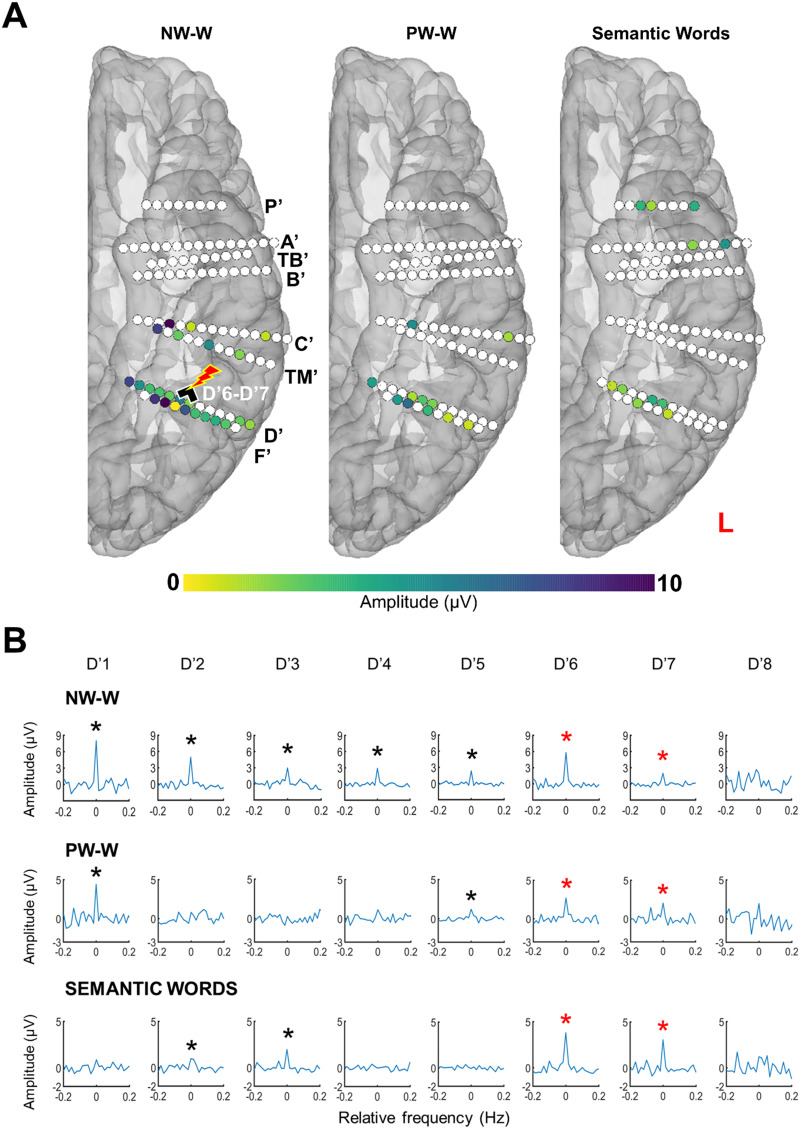
Intracerebral FPVS responses. (A) Spatial distribution of the baseline-corrected amplitude of word-selective responses recorded using continuous sequences of visual stimuli, either nonwords (NW) or pseudowords (PW), and of a semantic response recorded using the Semantic Word (SemWords) paradigm (see Figure 2). Each circle represents a single contact, colored circles correspond to significant contacts (*p* < 0.001; *z* score > 3.1, uncorrected), color-coded according to their response amplitude. The locations of these contacts are shown using a reconstructed cortical surface of SV’s brain. (B) Word-selective and semantic word responses recorded on electrode D′. The amplitude of these three responses (words over NW and words over PW for the Word paradigm, animals over cities for the Semantic Words paradigm) was quantified by summing the frequency of interest and the relative harmonics (i.e., Word paradigm: 1.2 Hz and 7 harmonics, up to 10.8 Hz excluding the 4th that coincided with the base frequency; SemWords: 1 Hz and 2 harmonics, up to 3 Hz). The number of harmonics included was based on the highest number of consecutive harmonics that showed a significant response across contacts (*z* score > 3.1; i.e., *p* < 0.001). The significance of a response was assessed for each response by the *z* score relative to the sum of the first harmonics before the base response (i.e., up to 6 Hz for the Word paradigm and 4 Hz for the Semantic Words paradigm). The 0 mark corresponds to the frequency of interest. * indicates significant responses at *p* < 0.001.

We measured electrophysiological word-selective responses on each contact with an FPVS Word paradigm including two conditions (NW-W, and PW-W) to identify different levels of visual word discrimination (Figure 4). Overall, we found 24 contacts (out of 174, 14%) with a significant response in at least one condition (Figure 4A). Critical contacts D′6–D′7 recorded large response amplitudes for both conditions. Among the few contacts showing significant responses in all conditions (*N* = 11), D′6 showed the sixth-largest response amplitude for NW-W and PW-W across all recorded contacts.

We also tested SV with an FPVS Word Semantic paradigm that provides responses relative to the semantic categorization of words (animals vs. cities; Volfart et al., 2021). D′6 and D′7 recorded large responses (second and fourth largest semantic response out of the 10 contacts that displayed a significant semantic response; Figure 4B), showing that this site was sensitive to word-based semantic memory, that is, to the meaning of the words. Altogether, these results show that the critical stimulation site inducing reading impairment (D′6–D′7) was located in a highly word-selective region, sensitive to whole-word forms beyond prelexical representations in one paradigm and to the meaning of words in the other paradigm.

### fMRI Word-Sensitivity of the Critical Stimulation Site

The fMRI word localizer experiment revealed a significant cluster of activation (*p* < 0.001 with Bonferroni correction) in the left occipito-temporal sulcus (Figure 1B) for visual word stimulation compared to rest (i.e., fixation cross; e.g., Ben-Shachar et al., 2007, 2011; Gaillard et al., 2006; Vigneau et al., 2005; Vinckier et al., 2007). This location is consistent with VWFA coordinates (Talairach space *x* = −43, *y* = −54, *z* = −12; Cohen et al., 2000). The coordinates of the voxel with the maximum activation are *x* = −44, *y* = −50, *z* = −16 in Talairach space, with a *Z* score of 10, *p* < 0.001 with Bonferroni correction.

The critical contact D′7 abuts this cluster of activation, being located at its anterior/medial edge (Figure 1B). To examine the word-sensitivity of D′7, we created masks with a 2 mm radius from the center coordinates of D′7. fMRI word-sensitive activations overlapped with D′7 (average *Z* score = 5.5; *p* < 0.001 uncorrected; *p* < 0.01 with Bonferroni correction).

### Effective Connectivity of the Reading Critical Site

Behavioral effects elicited by focal DES are known to be (partly) caused by the disturbance of connected brain regions beyond the stimulation site (Borchers et al., 2012; Jonas & Rossion, 2023). It is therefore important to highlight all brain regions affected by the stimulation to understand the critical networks underlying brain function and behavior, in the present case, reading functions. We used a recently developed original approach that employs DES, while concomitantly measuring frequency-tagged visually elicited neural activity across other brain regions (Angelini, Jacques, et al., 2024; Angelini, Volfart, et al., 2024).

**Figure 5.**
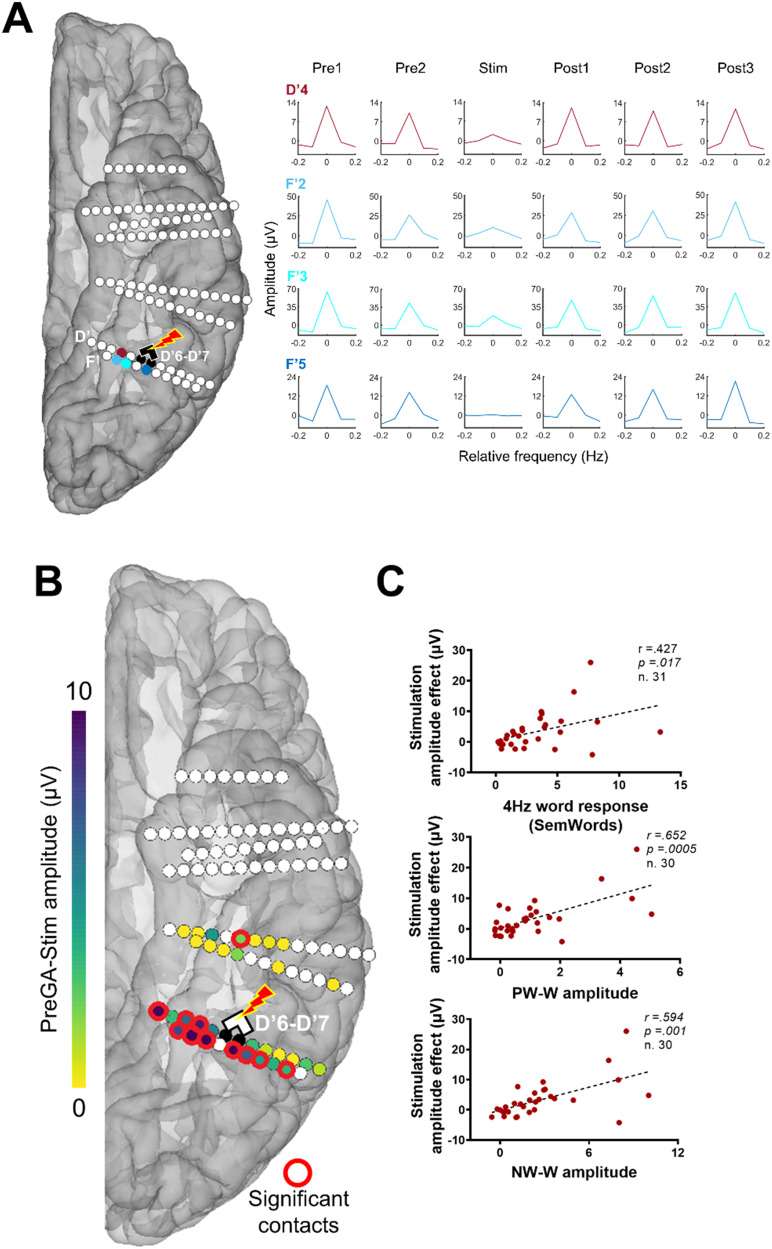
Effective connectivity of the critical site. (A) Examples of amplitude variation for the 2 Hz Semantic Words (SemWords2Hz) response with concurrent electrical stimulation of the critical contact D′6–D′7. Mean baseline-corrected FFT of the SemWords2Hz response across three stimulation sessions of contacts D′6–D′7 are shown for each period, before stimulation (Pre1, Pre2), during stimulation, and after stimulation (Post1, Post2, Post3). The locations of these contacts are shown on the left panel using a reconstructed cortical surface of subject SV’s brain. (B) Spatial distribution of the base response amplitude decreases during stimulation for each stimulated site displayed on a reconstructed cortical surface of subject SV’s brain. Contacts of interest are color-coded according to the baseline-corrected amplitude difference between the average of Pre1 and Pre2 and the stimulation periods (stimulation effect). Contacts with a significant difference are circled in red (*z* score > 2.32, *p* < 0.01). (C) Correlation plots between the stimulation amplitude effect for the base SemWords responses (baseline-corrected amplitude difference between the average of Pre1 and Pre2 and stimulation periods), and independent word responses (word-selective response computed outside stimulation), across the contacts of interest (*N* = 34 minus the 2 stimulated contacts). Outliers with values higher than *z* score = 3 were removed, and false discovery rate (FDR) corrections were applied to control for multiple comparisons (Benjamini & Hochberg, 1995). The Pearson correlation coefficient, the *p* values, and the number of contacts included in the analyses are indicated for each correlation.

To do so, we repeatedly administered electrical stimulation to the critical contacts D′6–D′7 concomitantly with the presentation of the SemWords2Hz paradigm (generating a response to words at 2 Hz). As indicated above, during these stimulation sessions, SV was asked to read aloud animal names (0.5 Hz), and her accuracy dropped as soon as the stimulation began (Figure 3). In addition to this behavioral effect, this procedure allowed us to record the real-time modulation of local and distant word responses caused by the stimulation. Since we did not test SV with the SemWords2Hz paradigm outside stimulation, we used the SemWords4Hz to select the pool of contacts included in the analysis. We found 32 contacts showing a significant 4 Hz word response in the SemWords4Hz paradigm recorded outside stimulation (34 contacts minus the 2 respective stimulated contacts, i.e., 32 contacts). Among these 32 contacts, some showed a strong reduction or a complete disappearance of the 2 Hz word response during D′6–D′7 stimulation (Figure 5). This effect was specific to the stimulation period and sometimes remained during the post-stimulation periods, with a gradual return to the prestimulation amplitude.

In total, 11 contacts (i.e., 34%) showed a significant decrease of the 2 Hz word response during the stimulation relative to the pre-stimulation period (average between Pre 1 and Pre 2 periods, see Materials and Methods). These contacts were located in the same structure as the stimulation site (OTS) and in the middle fusiform gyrus (medial and lateral parts; Figure 5B). One contact was located more anteriorly, in the anterior part of the collateral sulcus.

To determine whether this effect was functionally specific, we computed correlations between the size of the stimulation effect (difference of 2 Hz word response amplitude between the stimulation period and the average of Pre1 and Pre2 periods; see Figure 5A) and several independent functional responses acquired outside the stimulation sessions across the 32 corresponding nonstimulated contacts (Figure 5C). All correlations were computed by removing outliers (*z* score > 3), and FDR corrections were applied to control for multiple comparisons (Benjamini & Hochberg, 1995).

We found highly positive significant correlations between the size of the stimulation effect and the word-selective response amplitudes acquired for the two levels of discrimination (PW-W amplitude: *r*(28) = 0.652, *p* = 0.0005; NW-W amplitude: *r*(28) = 0.594, *p* = 0.001), showing that the more a contact was word-selective, the more this contact was affected by the stimulation (Figure 5C). The stimulation effect was also positively correlated with the 4 Hz word response acquired with the SemWord4Hz paradigm (*r*(29) = 0.427, *p* = 0.017). To evaluate the influence of physical measurements on the correlations relative to word selectivity, we conducted partial correlations using the Euclidean distance from the stimulation site and the amplitude of the stimulation artifact as control variables. These results showed that there was no significant influence of these two variables on the correlations between the stimulation effect and the PW-W/NW-W conditions, as the correlations remained significant once the influence of the control variables was considered (PW-W: *r* = 0.62, *p* = 0.001; NW-W: *r* = 0.53, *p* = 0.004).

## DISCUSSION

We report a comprehensive behavioral and neural investigation of a case of transient reading impairment due to DES of the word-selective left OTS. To put things in perspective, while DES to temporal lobe structures is regularly performed to assess the integrity and lateralization of language in our clinical unit (around 35 SEEG explorations per year), with a majority of patients implanted in left temporal lobe structures, this is one of the rare cases of selective transient reading impairment observed in almost 20 years of clinical investigations. In contrast, transient impairments in naming are regularly observed following DES to the ventral region of the anterior temporal lobe (baso-temporal area of language [BTLA]; Abdallah et al., 2021; Bédos Ulvin et al., 2017; Lüders et al., 1991), perhaps due in part to the more frequent implantations in the anterior temporal lobe than posterior regions of the VOTC. Overall, the present observations in an original case strengthen the role of the left VOTC in reading, showing its criticalness as a cortical node of a visual language network.

Compared to previous reports (Hirshorn et al., 2016; Mani et al., 2008; Sabsevitz et al., 2020; Woolnough et al., 2021, 2022; Woolnough & Tandon, 2024), the present case has many strengths, making it, to our knowledge, the clearest reported case of pure alexia due to DES to date.

First, the transient reading impairment observed during DES was clear, immediate, reproducible on different days of testing, and concerned all aspects of letter/word reading. While previous studies reported results for a few trials or behavioral tasks, we were able to assess behavioral performance in a variety of reading tasks, often with both accuracy rates and response times. In doing so, we highlighted a selective reading deficit, with no impairment in other cognitive functions, especially in picture naming. SV experienced transient impairments in reading isolated words, both with and without time constraints, as well as in reading text. She would stop or slow down, explaining she “couldn’t make the word,” but recognized the unread word after stimulation. She was also impaired without having to read aloud in written lexical decision tasks, excluding a deficit due to phonological access. She also struggled reading syllables and pseudowords (nonsignificant decrease of accuracy for the 2 tasks, significant increase on RT for syllables). Finally, we challenged her letter-by-letter reading performance by increasing the presentation rate of isolated letters. While SV was able to accurately name the letters presented within a word without time constraint, she was impaired with fast isolated letter presentations. These observations suggest that speeding up the presentation of letters weakened her ability to link letters to the phonology of their names. Alternatively, the patient might have been impaired at visually identifying the letters presented at a fast(er) rate during DES. However, this alternative possibility is unlikely given that (1) each letter was still presented for a relatively long time (150 ms), (2) the patient’s ability to read letters at a slow rate, (3) her intact self-reported percept of letters, and (4) the relative anterior location of the stimulated site within the VOTC.

Second, the transient impairment was selective to reading, without anomia, aphasia, agraphia, or alterations in semantic functions and object recognition deficit being reported during DES. Writing words to dictation was also unimpaired, as usually described in pure alexia (e.g., Dejerine, 1892; Purcell et al., 2014; Sabsevitz et al., 2020; Starrfelt & Shallice, 2014), showing the critical role of the targeted region for orthographic input but not production processes. While picture identification is sometimes impaired in pure alexia for images of high visual complexity (e.g., Behrmann et al., 1998), suggesting a more general visual processing deficit (e.g., Behrmann et al., 1998; Farah & Wallace, 1991, cited in Starrfelt & Behrmann, 2011), there was no semantic deficit during (picture) semantic matching here. Finally, despite the limited testing of number reading (only one stimulation, no rapid serial presentation of single numbers), our findings show that reading numbers was not impacted by stimulation. This does not align with observations suggesting that number reading may be affected in pure alexia (Leff et al., 2006; Starrfelt & Behrmann, 2011) and goes against the view that word and number reading share common cognitive and neural mechanisms without clear separation (Starrfelt & Behrmann, 2011, for a review of dissociation and nondissociation studies). To the contrary, they support the view of cognitive and neural dissociation between reading words/letters and reading Arabic numbers (e.g., Carreiras et al., 2015; Dotan & Friedmann, 2019; Hannagan et al., 2015; see also Cipolotti, 1995, for a dissociation case in dyslexia).

Overall, the reading impairment during DES was found for all types of letter strings, suggestive of a deficit at orthographic input levels affecting both word recognition and the conversion of graphemes into phonemes. In a cognitive perspective (e.g., DRC; Coltheart et al., 2001), the deficit either affects both the direct lexical and the sublexical reading routes, or it stems from an earlier stage, where print is identified as an abstract letter identity (i.e., recognizing the identity of the letter “F”). This latter possibility is unlikely as discussed above, otherwise she would have been unable to read letter by letter.

**Figure 6.**
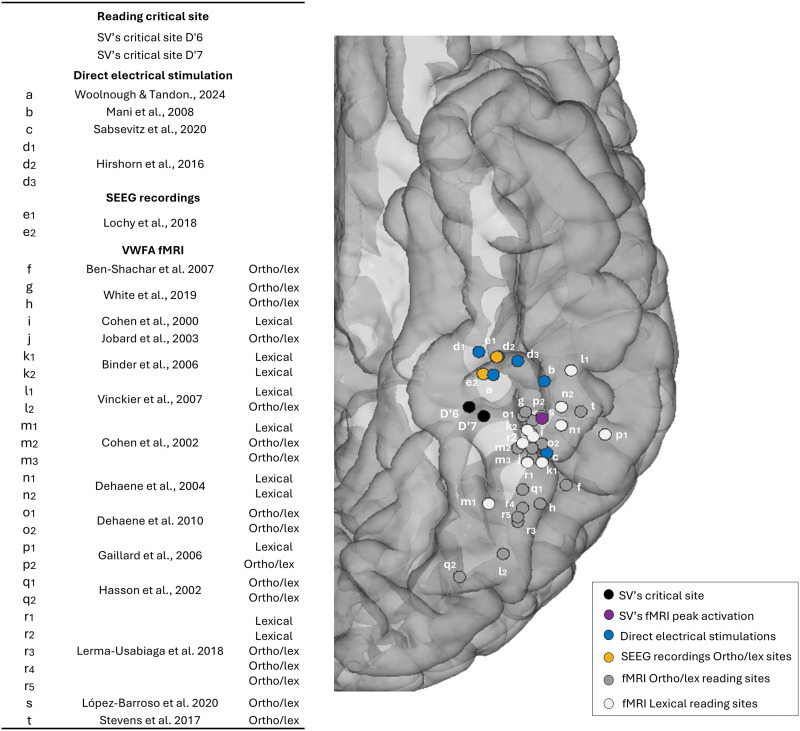
Spatial relationship between D′6–D′7 critical site, previous fMRI visual word form area coordinates, previous direct electrical stimulation critical sites and previous stereotactic electroencephalography (SEEG) word-selective responses. These sites are displayed in the Talairach space using a reconstructed cortical surface of the Colin27 brain: SV’s critical site for reading (black), SV’s fMRI word sensitive peak activation (purple), electrical stimulations sites inducing alexia (blue), prelexical and lexical peaks of amplitude recorded in SEEG using the same fast periodic visual stimulation reading paradigm as here (yellow, Lochy et al., 2018), fMRI orthographic/lexical sites (gray), and fMRI lexical sites (white) extracted from Caffarra et al. (2021). MNI coordinates were transformed to Talairach with an online transformation tool: https://bioimagesuiteweb.github.io/webapp/mni2tal.html.

The pair of electrode contacts associated with the DES-evoked reading impairment falls in a word-selective region as measured with FPVS paradigms, lying at the anterior edge (*y* = −49) of the typical location of the VWFA (e.g., Cohen et al., 2000: *x* = −43, *y* = −54, *z* = −12 in Talairach space; the y-coordinate ranges from ≈ −54 to −42 across studies; Caffarra et al., 2021; Jobard et al., 2003; Martin et al., 2015; Olulade et al., 2013; Figure 6). Despite this anterior location, the critical sites D′6 and D′7 recorded large iEEG word-selective responses (prelexical and lexical). Interestingly, and more consistently with their anterior location, these two lexical contacts also presented the most significant response amplitudes during a written word semantic categorization paradigm, making them sensitive both to the lexical representation of words (selective responses to words among pseudowords) and the meaning of words (selective responses to words of animals among words of cities). Overall, DES to critical reading sites (including the present study) and prelexical and lexical responses recorded by iEEG are located anteriorly to most fMRI activations (Figure 6), leaving open the possibility that fMRI usually misses a large part of critical reading regions, probably because of magnetic susceptibility artifacts (Wandell, 2011; Yeatman et al., 2013).

At first glance, the finding of a critical reading site, sensitive to prelexical, lexical, and word semantic representations, located rather anteriorly to most fMRI activations, does not align with a deficit in the visual analysis of letters or letters strings, which is thought to happen posteriorly in the VWFA according to a hierarchical view of reading (Dehaene et al., 2005). Admittedly, given that the effect of a DES can also disrupt the functioning of connected regions (Angelini, Jacques, et al., 2024; Angelini, Volfart, et al., 2024; Borchers et al., 2012; Desmurget et al., 2013; Jonas & Rossion, 2023; Penfield, 1958), we cannot exclude that both anterior and posterior regions in the VOTC were affected. The view of a specific single region processing whole words is largely debated (e.g., see Caffarra et al., 2021, for review), and an alternative functional organization of the left VOTC for reading as a “collection of multiple discrete subregions” (Caffarra et al., 2021, p. 3057; White et al., 2019, 2023; see Caffarra et al., 2021, for review) with different functional connections has been proposed (e.g., White et al., 2019; Yablonski et al., 2024; Yeatman & White, 2021). The hypothesis of two subregions within the original VWFA, with distinct functional connections (Yablonski et al., 2024; Yeatman & White, 2021; see Caffarra et al., 2021, for review) proposes a posterior region sensitive to visual features (VWFA-1) and a more anterior one (VWFA-2) connected with higher level language processes (Yablonski et al., 2024), reminiscent of the orthography-to-semantic interface area that had been proposed in cognitive neuropsychology (Purcell et al., 2014). In our case, we could consider that the stimulated region may be highly and specifically connected with multiple areas critical for reading given that the DES effect propagated to these different regions, explaining the wide range of reading deficits (lexical, prelexical, and letter).

Here, for the first time to our knowledge, we took advantage of a recently developed approach (Angelini, Jacques, et al., 2024; Angelini, Volfart, et al., 2024) to investigate the effective connectivity of a reading critical site using DES coupled with frequency tagging. We observed that DES affected local and remote (anterior) word responses. This anterior remote effect suggests the reading deficit observed here could, at least partially, be related to the disruption of a region located slightly anteriorly to the VWFA, at the interface of lexical orthography and semantics (Purcell et al., 2014). Unfortunately, due to the absence of occipital electrodes, we were unable to assess the connectivity of the critical site with posterior regions, especially those involved in letter processing. Nevertheless, we showed that the stimulated effect is not restricted to the stimulated site and that DES affected multiple regions in the left VOTC. Another, not mutually exclusive, possibility is that the stimulated region processes both prelexical and lexical representations and, to a lesser degree, is also involved in letter identification. This is in line with iEEG evidence of intermingled neuronal populations for letters, prelexical and lexical processes in the same region of the left middle fusiform gyrus (Lochy et al., 2018). Unfortunately, SV was not tested with an FPVS condition to record letter-selective responses (i.e., letters vs. pseudocharacters; see Lochy et al., 2015, 2018).

In conclusion, we report an original DES case of pure alexia associated with wide range of reading deficits (lexical, prelexical, and letter), potentially reflecting the complex intrinsic connectivity between multiple subregions within the word-selective left VOTC during DES, supporting the role of this region as a critical node of a reading cortical network.

## ACKNOWLEDGMENTS

We thank subject SV for her involvement and willingness to participate in the study.

## FUNDING INFORMATION

Bruno Rossion, European Research Council (https://dx.doi.org/10.13039/501100000781), Award ID: HUMANFACE 101055175. Aliette Lochy, Fonds National de la Recherche du Luxembourg, Award ID: FNR-CORE C21/SC/16241557/READINGBRAIN.

## AUTHOR CONTRIBUTIONS

**Marion Marchive**: Conceptualization: Equal; Data curation: Equal; Formal analysis: Equal; Investigation: Equal; Methodology: Equal; Writing – original draft: Lead; Writing – review & editing: Lead. **Luna Angelini**: Conceptualization: Equal; Data curation: Equal; Formal analysis: Equal; Investigation: Equal; Methodology: Equal; Writing – original draft: Equal; Writing – review & editing: Equal. **Aliette Lochy**: Conceptualization: Equal; Investigation: Supporting; Supervision: Supporting; Writing – original draft: Equal; Writing – review & editing: Equal. **Louis Maillard**: Methodology: Supporting; Resources: Supporting; Supervision: Supporting; Writing – review & editing: Supporting. **Sophie Coulnat-Coulbois**: Investigation: Supporting; Methodology: Supporting; Resources: Supporting; Supervision: Supporting; Writing – review & editing: Supporting. **Bruno Rossion**: Conceptualization: Equal; Data curation: Equal; Funding acquisition: Lead; Investigation: Equal; Methodology: Equal; Resources: Equal; Supervision: Equal; Validation: Equal; Writing – original draft: Equal; Writing – review & editing: Equal. **Jacques Jonas**: Conceptualization: Equal; Data curation: Equal; Funding acquisition: Equal; Investigation: Equal; Methodology: Equal; Supervision: Equal; Validation: Equal; Writing – original draft: Equal; Writing – review & editing: Equal.

## DATA AND CODE AVAILABILITY STATEMENTS

The anonymized behavioral and SEEG spreadsheet data are available on the Open Science framework: https://osf.io/vdwna/overview?view_only=7774becfb4c74e7689f016b3942fdcb3. Due to institutional restrictions, the anonymized raw data are available upon request from the corresponding author.

## Supplementary Material








