# Supplementary material for: Intracerebral Electrical Stimulation of the Left Occipito-Temporal Cortex Induces Pure Alexia
Source: Neurobiol Lang (Camb). 2025 Dec 18;6:NOL.a.205. doi: 10.1162/NOL.a.205 (PMC12727048; doi:10.1162/NOL.a.205)
Supplement: Supplementary file 1 [file nol-6-1-205-s001.pdf]

Supplementary materials

Reading test from ECLA16+	Instruction	Accuracy (score – errors)	Time (seconds)
Verbal fluency	*Name as many animals as you can in one minute*	13	60
L'Alouette<br>(text without meaning)	*Read aloud as best you can a meaningless text in 3 minutes*	257	110
RAN picture (rapid automatic naming)	*Name as fast as possible, without errors, all images*	25	25
Letter naming	*Name as fast as possible, without errors, all letters*	48	22
Le pollueur<br>(text with meaning)	*Read a text as correctly and quickly as possible in one minute*	148	60
Reading lists of 20 isolated words (20 irregular, 20 regular and 20 pseudo-words)	*Read as correctly and quickly as possible all words presented (time recorded per list)*	Regular : 20<br>Irregular : 17<br>Pseudo-words : 18	Regular :16<br>Irregular : 16<br>Pseudo-words : 20

Table 1. Description of each task from ECLA 16+ with instructions*. The name of the reading tests and instructions are described. SV's performance is indicated for each task with accuracy (score – errors) and response times (seconds).*

Tasks	Stimulation parameters				Accuracy		
	Contact	*Trial*	*Intensity (mA)*	*Time (sec)*	*before*	*during*	*after*
Reading aloud isolated words	D'1-2	1	1	5	4/4	4/4	0/0
	D'3-4	1	1.2	5	4/4	4/4	3/3
	D'4-5	1	1.2	5	3/3	5/5	3/3
	D'5-6	1	1.2	5	4/4	4/4	1/1
	D'7-8	1	1.2	5	4/4	5/5	2/2
	F'1-2	1	1.2	5	4/4	4/4	2/2
	F'2-3	1	1.2	5	4/4	4/4	2/2
	F'3-4	1	1.2	5	4/4	4/4	3/3
	F'5-6	1	1.2	10	3/3	9/9	3/3
	F'6-7	1	1.2	5	4/4	5/5	4/4
	TM'3-4	1	1.2	10	4/4	4/4	3/3
Reading aloud isolated words	D'6-D’7	5	3 (1.2 mA), 1 (1.6mA), 1 (1.8mA)	1 (5sec), 4 (10sec)	16/16	11/19	37/39
Reading syllables		1	1,2	10	3/3	5/6	5/5
Reading pseudo-words		1	1,2	10	3/3	2/4	3/3
Reading a text		1	1,2	10	71/72	3/5	69/70
DLT (paper)		1	1,2	10	4/4	2/3	4/4
Reading fast letters (computerized)		3	1,8	10	51/52	17/52	43/52
DLT (computerized)		2	1,8	10	37/37	4/5	32/32
Reading words in FPVS		3	1,2	10	28/34	8/15	46/62

***Table 2*. *All electrical stimulation for the reading tasks performed*.** *For each task, stimulation parameters are described with the number of stimulation sessions, the intensity and the time (seconds) performed on each contact. The accuracy is also indicated before, during and after stimulation sessions.*

Tasks	Accuracy				RT		
	outside	*during*	*x²*	*p value*	*outside*	*during*	*p value*
Reading isolated words	53/55	11/19	17.881	**<.001**	774.906	1174.545	**0.002**
Reading syllables	8/8	5/6	1.436	0.231	515	992	**0.007**
Reading pseudo-words	6/6	2/4	3.75	0.053	800	1360	0.285
DLT (paper)	8/8	2/3	1.556	0.212	1040	2280	**<.001**
DLT (computerized)	69/69	4/5	13.989	**<.001**	88.449	1638.75	**0.02**
Reading number	4/4	5/5	/	/	1235	1552.00	0.314
Reading a text	140/142	3/5	27.175	**<.001**	/	/	
Reading fast letters (computerized)	94/104	17/52	49.111	**<.001**	/	/	
Reading words in FPVS	74/96	8/15	3.791	0.052	/	/	
Visual object naming	24/24	21/21	/	/	1325	1510.476	0.366
Auditory naming	15/15	6/6	/	/	1389.33	1280	0.500
Auditory lexical decision task	8/8	5/5	/	/	1075	960	0.690
Writing	8/8	2/2	/	/	2765	3660	0.285
Semantic matching	8/8	4/4	/	/	1480	1700	0.514

Table 3. Statistical analyses of accuracy (outside vs during) and response times (two-tailed permutation test) for each task on D'6-D'7 stimulation. *For each task, performance outside stimulation (average of before and after) and during stimulation were statistically compared for accuracy and response times (RTs).*

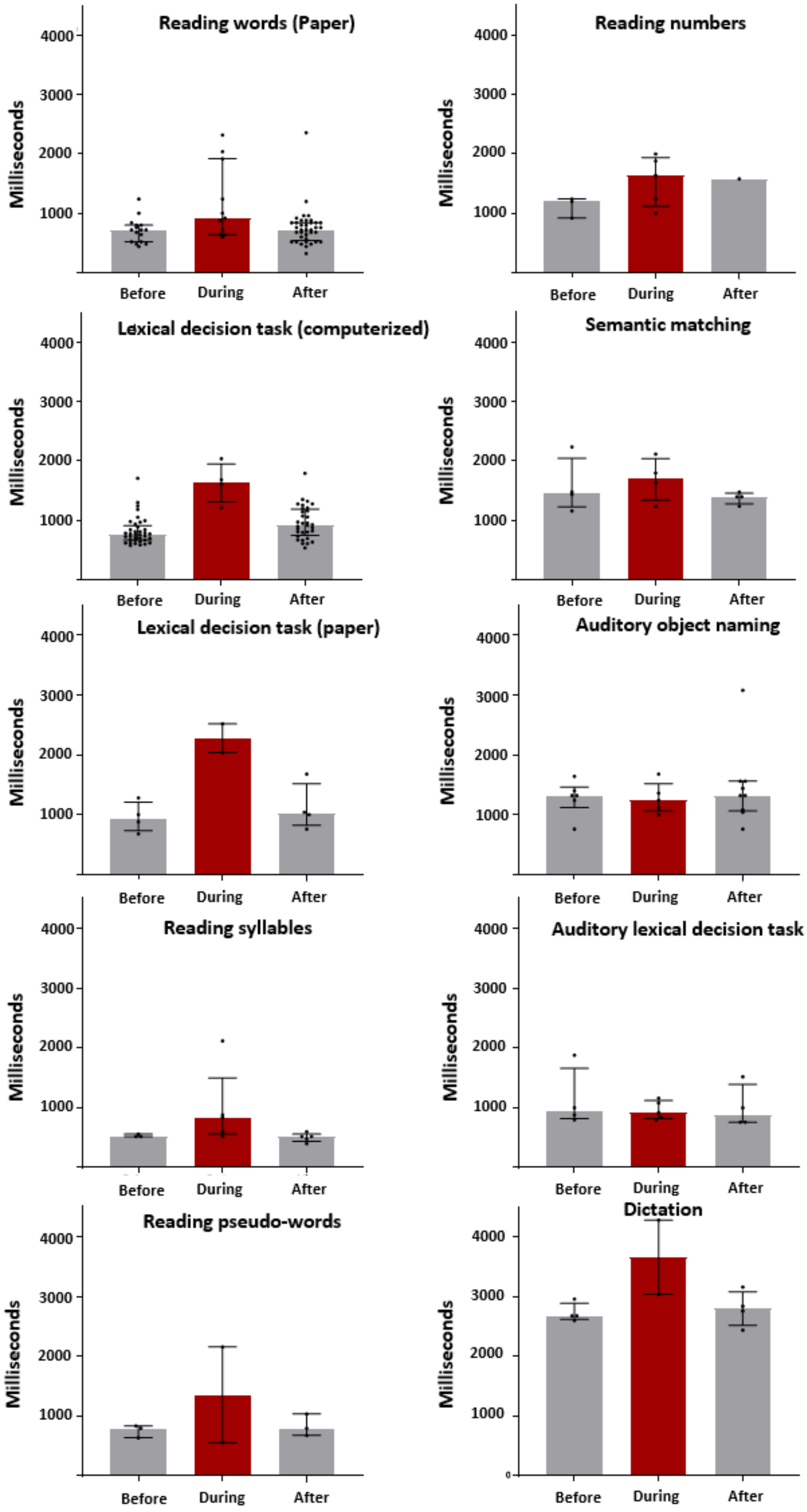
Reading words (Paper)
Reading numbers
Lexical decision task (computerized)
Semantic matching
Lexical decision task (paper)
Auditory object naming
Reading syllables
Auditory lexical decision task
Reading pseudo-words
Dictation
Milliseconds
1000
2000
3000
4000
0
Before
During
After

Figure 7. Summary of the electrical stimulations performed on contacts D'6-D'7 for the reading tasks represented with median, interquartile range and single-trial data. *Response times in milliseconds measured before, during (in red), and after stimulation. The graph displays the median response times for each condition, with error bars indicating the interquartile range. Single-trial data points are also plotted.*

Video 1. Direct electrical stimulation of critical reading contacts during a reading-aloud task with animal names. *Performance of SV before, during, and after stimulation of critical reading sites during a reading-aloud task involving animal names presented periodically every 4 images among city names. She was instructed to raise her hand whenever she experienced difficulty in reading. In the second part of the video, SV provides a subjective description of the effect.*

Video 2. Direct electrical stimulation of critical reading contacts during a reading-aloud of isolated words. *Performance of SV before, during, and after stimulation of critical reading sites during a reading-aloud task involving isolated words on paper. Correct responses are marked with a green check, while unread words are marked with red crosses. Unread words are spelled out letter by letter. In the second part of the video, SV provides a subjective description of the effect.*

Video 3. Direct electrical stimulation of critical reading contacts during a reading-aloud of isolated letters in a Rapid Serial Visual Presentation. *Performance of SV before, during, and after stimulation of critical reading sites during a fast presentation task involving reading aloud isolated letters. Correct responses are marked with a green check, while unread words are marked with red crosses. In the second part of the video, SV provides a subjective description of the effect.*
